# Proteomic Analysis on Sequential Samples of Cystic Fluid Obtained from Human Brain Tumors

**DOI:** 10.3390/cancers15164070

**Published:** 2023-08-11

**Authors:** Lorenzo Magrassi, Francesca Brambilla, Raffaello Viganò, Dario Di Silvestre, Louise Benazzi, Giuseppe Bellantoni, Gian Marco Danesino, Sergio Comincini, Pierluigi Mauri

**Affiliations:** 1 Neurosurgery, Dipartimento di Scienze Clinico-Chirurgiche e Pediatriche, Università degli Studi di Pavia, Fondazione IRCCS Policlinico S. Matteo, 27100 Pavia, Italy; 2 Istituto di Genetica Molecolare—CNR, 27100 Pavia, Italy; 3 Proteomics and Metabolomics Institute for Biomedical Technologies (ITB-CNR), Segrate, 20090 Milan, Italy; francesca.brambilla@itb.cnr.it (F.B.); raffaello.vigano@itb.cnr.it (R.V.); dario.disilvestre@itb.cnr.it (D.D.S.); louise.benazzi@itb.cnr.it (L.B.); pierluigi.mauri@itb.cnr.it (P.M.); 4 Struttura Complessa di Neurochirurgia, Fondazione IRCCS Policlinico S. Matteo, 27100 Pavia, Italy; g.bellantoni@smatteo.pv.it; 5 Struttura Complessa di Radiologia Diagnostica per Immagini 2—Neuroradiologia, Fondazione IRCCS Policlinico S. Matteo, 27100 Pavia, Italy; gm.danesino@smatteo.pv.it; 6 Dipartimento di Biologia e Biotecnologie, Università di Pavia, 27100 Pavia, Italy; sergio.comincini@unipv.it

**Keywords:** tumor cyst, cystic fluid, secretory meningioma (SM), cystic schwannoma (CS), glioblastoma (CG), proteomics

## Abstract

**Simple Summary:**

Cystic brain tumors are a heterogeneous category comprising a wide range of lesions both intra- and extracerebral. While the presence of large cysts is hallmark of rare benign tumors, such as craniopharingiomas, hemangioblastomas and secretory meningiomas, cysts may less frequently develop into 8th nerve schwannomas and high-grade gliomas. The mechanisms leading to cyst formation have not been clarified. The most commonly accepted hypothesis indicates an increased vascular permeability with serum protein accumulation as the possible culprit. In order to clarify the composition of cystic fluid and its possible variations in different tumors, we performed an untargeted proteomic analysis of cystic fluid serially sampled by mini-invasive methods from secretory meningioma (SM), cystic schwannoma (CS) and cystic high-grade glioma (CG). Our data indicate that proteins contained in the cystic fluid of those tumors remains stable over time. Together with proteins originating from serum and cerebrospinal fluid (CSF) that were common to all cystic fluids examined, we identified proteins whose presence was restricted to specific tumors. Furthermore, applying a protein–protein interactions network analysis and an overrepresentation evaluation, we were able to identify functional pathways that were typical for each of the three tumor histotypes we studied. Our results demonstrate that the cystic fluid sampled from different tumors contains proteins derived from multiple sources: serum, CSF and the tumor itself, indicating that the proteomic study of this tumor component, which is often neglected, may help us to clarify tumor–host tissue interactions with potential diagnostic and therapeutic implications.

**Abstract:**

Cystic formation in human primary brain tumors is a relatively rare event whose incidence varies widely according to the histotype of the tumor. Composition of the cystic fluid has mostly been characterized in samples collected at the time of tumor resection and no indications of the evolution of cystic content are available. We characterized the evolution of the proteome of cystic fluid using a bottom-up proteomic approach on sequential samples obtained from secretory meningioma (SM), cystic schwannoma (CS) and cystic high-grade glioma (CG). We identified 1008 different proteins; 74 of these proteins were found at least once in the cystic fluid of all tumors. The most abundant proteins common to all tumors studied derived from plasma, with the exception of prostaglandin D2 synthase, which is a marker of cerebrospinal fluid origin. Overall, the protein composition of cystic fluid obtained at different times from the same tumor remained stable. After the identification of differentially expressed proteins (DEPs) and the protein–protein interaction network analysis, we identified the presence of tumor-specific pathways that may help to characterize tumor–host interactions. Our results suggest that plasma proteins leaking from local blood–brain barrier disruption are important contributors to cyst fluid formation, but cerebrospinal fluid (CSF) and the tumor itself also contribute to the cystic fluid proteome and, in some cases, as with immunoglobulin G, shows tumor-specific variations that cannot be simply explained by differences in vessel permeability or blood contamination.

## 1. Introduction

Cystic change in solid human parenchymal primary brain tumors is a relatively rare event and, at least, mechanistically, cyst formation in these tumors is still not fully understood. Obvious exceptions to this rule are represented mainly by cerebellar pilocytic astrocytomas, pleomorphic xanthoastrocytomas, secretory meningiomas and hemangioblastomas that are often linked to a large accompanying cyst. These intracranial tumors that often develop one or more cysts are infrequent, and altogether they represent less than 10% of all primary brain tumors. A significant intratumoral cystic component occurs in 7–23% of glioblastomas and grade IV astrocytomas [1], and the presence of a large cyst is usually associated with a younger age and longer survival independent of treatments [1]. Furthermore, cystic fluid from gliomas, meningiomas and schwannomas may stimulate proliferation and/or migration of stable human cell lines [2].

Brain tumor cyst fluid has been considered secondary to a transudative process from plasma [3]. The most widely supported hypothesis of cystic fluid formation in these tumors considers that local disruption of the blood–brain barrier (BBB) leads to exudation of plasma from blood vessels leading to plasma protein accumulation followed by passive accumulation of interstitial fluid that favors micro- and macrocyst formation [4,5,6]. This hypothesis is supported by the finding of a higher level of VEGF in macrocystic cerebellar hemangioblastomas [7], gliomas [8,9] and adamantinomatous craniopharyngiomas [10] compared to tumors of the same histotypes with a solid pattern.

In general oncology, a distinction is usually made between pseudocysts that are filled with fluid passively transported into the lumen (e.g., cerebrospinal fluid percolation) and true cysts where fluid accumulates by osmotic/secretory or active transport mechanisms in the absence of significant tumor degeneration [11]. In practice, this distinction is often difficult to make on neurosurgical specimens. A more useful classification with important neurosurgical implications distinguishes between intratumoral cysts and peritumoral cysts, such as in cerebellar hemangioblastomas and pilocytic astrocytomas. In peritumoral cysts, which are induced but not infiltrated by the tumor, the cystic walls do not require resection once the solid portion of the tumor has been removed and the fluid content aspirated [12,13]. Very often, the cytological evaluation of the cystic fluid obtained from primitive brain tumors do not show tumoral cells, therefore, the result of the analysis is nondiagnostic [14].

There is a long history of biochemical and immunological studies on the cystic fluid of primary brain tumors [3]. However, to the best of our knowledge, data resulting from proteomic analysis of brain tumor cystic fluid are still limited. After two initial proteomic studies on hemangioblastoma cystic fluid using two-dimensional gel electrophoresis [6,15], other investigators characterized, using liquid chromatography coupled to a tandem mass spectrometry (LC-MS/MS), the cystic fluid of adamantinomatous craniopharyngioma [16] and glioblastoma and described their protein composition. However, all these studies were performed on samples collected during the initial surgery, and when multiple samples of brain tumor cystic fluid were obtained, they were not submitted to proteomic studies [17]. Moreover, we were unable to find studies on multiple samples obtained from the same lesion in the same patient at different times before treatments.

We performed a comparative proteomic work analyzing the cystic fluid of a secretory meningioma (SM), a cystic schwannoma (CS) and a cystic high-grade glioma (CG) obtained at multiple time points. The samples were collected either by direct percutaneous puncture of the tumoral cyst or through an implanted catheter connected to an Ommaya reservoir. Fluid collection was performed when the patient became symptomatic and, in all cases, the fluid collected was submitted to proteomic analysis with an LC-MS/MS followed by label-free quantification, including extraction of differential expressed proteins (DEPs) and evaluation of involved pathways [18].

We found that independently from the histotypes, the most abundant proteins were derived from plasma. However, cerebrospinal fluid (CSF) marker proteins, such as prostaglandin-H2 D-isomerase (also known by clinicians as Beta-trace protein) were also present together with tumor-specific proteins. Our results suggest that although plasma leaking from local BBB disruption is an important contributor to cyst formation, other mechanisms, like CSF percolation through the tumor and secretion or protein shedding from the tumor, also play a role in cystic fluid formation.

## 2. Materials and Methods

### 2.1. Patients

All patients were treated at the Fondazione I.R.C.C.S. Policlinico San Matteo between 2018 and 2020; all patients signed an informed consent authorizing the use of their anonymized clinical data for biochemical analysis and clinical research. A synopsis of the history of each patient follows.

**Figure 1:**
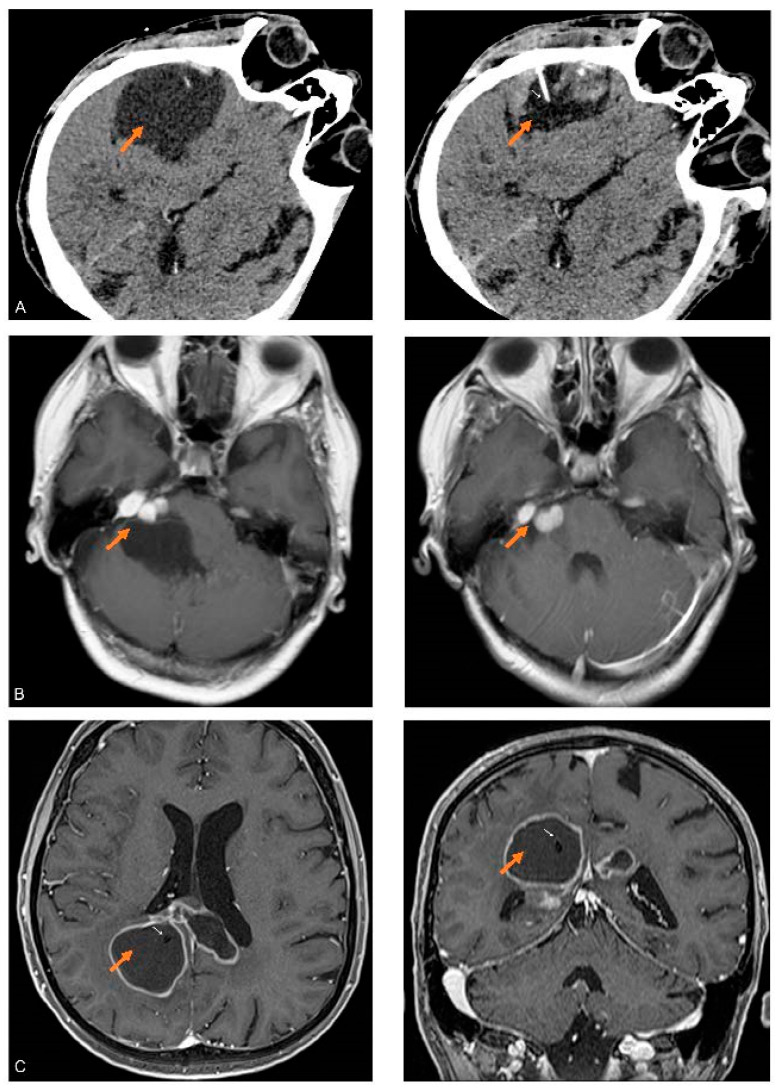
CT and MRI images of the cystic brain tumors studied: (**A**) Axial CT image of the SM obtained before (**left**) and after partial aspiration (**right**) of fluid in the cyst (orange arrows). In the right image, the needle (white arrow) used for the aspiration of the fluid is visible in the shrunken cystic cavity. (**B**) Axial MRI T1 weighed images with contrast, obtained before (**left**) and after (**right**) partial aspiration of fluid in the cyst (orange arrows) associated with the right-sided CS. (**C**) Axial and coronal MRI T1 weighed images with contrast of CG after the implantation of a catheter (white arrows) inside the largest cyst (orange arrows).

Patient 1 (male, 77 years): Twelve years before the first collection of cystic fluid, the patient was submitted to the surgical removal of a largely cystic fronto-temporal secretory meningioma (SM), a subcentimetric residual of the lesion was left, since it resulted tightly attached to one of the frontal rami of the middle meningeal artery. The residual lesion remained stable up to one year before the first collection of cystic fluid, when a follow-up MRI (Figure 1A) demonstrated the development of a small multiloculated cyst associated with the tumor residual. At that time, the patient was asymptomatic and refused further surgery. In the following months, the patient started to complain of headaches without any neurological deficit; a CT scan indicated that the cystic component of the relapsing meningioma was enlarged, but the patient accepted only a minimally invasive approach consisting in the percutaneous puncture of the tumoral cyst followed by the aspiration of the cystic fluid to relieve symptoms. This procedure was repeated three times at intervals of three months and was always followed by a rapid improvement in the clinical condition of the patient. In the months after the third aspiration, the patient’s conditions, again, became progressively worse for the relapsing enlargement of the tumoral cyst, and the patient accepted to be submitted to a new surgical removal of the tumor and the cyst.

Patient 2 (female, 51 years): Eight years before the first collection of cystic fluid, the patient was submitted for the surgical removal of a large right schwannoma of the 8th cranial nerve without any cystic component using a retrosigmoid approach. A small residual of the lesion tightly attached to the 7th cranial nerve was left in place to avoid any damage to the nerve. The patient was lost at follow-up until two weeks before the first collection of cystic fluid, when she spontaneously returned lamenting gait instability and headache. A contrast enhanced MRI showed a modest enlargement of the tumor residual and a large multiloculated cyst that starting from the tumor developed into the right cerebellar hemisphere (Figure 1B). The patient accepted only a minimal invasive approach consisting in fluid aspiration to relieve symptoms. The first puncture was successful with complete relief of the signs and symptoms. A relapse of the sign and symptoms occurred after a month. The patient was again treated with percutaneous cystic aspiration, again with temporary relief of the symptoms. After further relapse of the symptoms due to the enlargement of the cyst, the patient accepted a limited surgical approach with open fenestration of the cyst followed by stereotactic radiosurgery on the residual tumor.

Patient 3 (male, 65 years): Approximately a decade after the diagnosis of a macroprolactinoma successfully treated by chronic administration of cabergoline, the patient developed headaches, dizziness and generalized hyposthenia followed by objective walking difficulties after two weeks. A contrast MRI revealed a large bilateral cystic lesion with a thin contrast enhancing periphery involving the body and splenium of the corpus callosum together with the adjacent parietal and cingulate lobes (Figure 1C). The patient was submitted to stereotactic biopsy and implantation of a catheter into the cyst, the catheter was connected to a subcutaneous reservoir. The result of the biopsy was compatible with high-grade glioma. After two successful taps of the reservoir separated by three weeks, the catheter occluded, and no further sampling of the cystic fluid was possible; considering the stability in the dimensions of the cyst, the catheter was not substituted. The patient was treated with chemotherapy (temozolomide) and radiation therapy with stabilization of the mass, and a relapse occurred 15 months after the initial biopsy when a distant solid parenchymal mass developed at the level of the right temporal lobe.

Overall, three samples of cystic fluid separated by three months were obtained from patient 1, and two samples separated by one month were obtained from patient 2, and two samples separated by 3 weeks were obtained from patient 3.

### 2.2. Sample Preparation and LC-MS/MS Analysis

Cystic fluids, maintained as aliquots at −80 °C, were first clarified using centrifugation at 13,000 rpm for 10 min. Pellets were discarded, and the fluids were diluted 1:50 with water (LC-MS grade) to quantify the protein content using a Qubit Protein assay kit on a QubitTM4 Fluorometer (Invitrogen, Thermo Fisher Scientific, Waltham, MA, USA); 50 μg of proteins were recovered from SM, CS and CG fluids and processed using the EasyPep MS Sample Preparation Kit (Thermo Fisher Scientific, Waltham, MA, USA), according to the kit’s protocol. Each sample was analyzed in two technical replicates using the LC-MS/MS platforms: Eksigent nanoLC-Ultra 2D System (Eksigent, part of AB SCIEX, Dublin, CA, USA) for nano liquid chromatography coupled with an LTQ Orbitrap XLTM ETD or Surveyor HPLC coupled to an LTQ mass spectrometer (Thermo Fisher Scientific, San Jose, CA, USA) for MS/MS analyses. In particular, peptides were separated with the following eluent gradient: (A) 0.1% formic acid in water; (B) 0.1% formic acid in acetonitrile; the gradient profile was 5–60% B in 110 min, 60–95% B in 15 min; peptides were analyzed using tandem MS following the previously reported methods [19,20].

### 2.3. Extraction of Differentially Expressed Proteins

The experimental tandem mass spectra (MS/MS) produced using LC-MS/MS analysis were matched against the in silico tryptic peptide sequences of the Homo sapiens protein database (74,842 entries) retrieved from UNIPROT (https://www.uniprot.org/help/publications (accessed on 1 March 2021) The version of UniprotKB Homo Sapiens proteome used in the study was the 2021_01 release, updated on 10 February 2021. Data processing was performed with Proteome Discoverer 2.1 software (Thermo Fisher Scientific, Waltham, MA, USA), based on the SEQUEST algorithm [21]. The matches among spectra were only retained if they had a minimum Xcorr of 2.0 for +1, 2.5 for +2 and 3.5 for +3 charge state. Percolator node was used with a target–decoy strategy to provide a final false discovery rate at a peptide spectrum match (PSM) level of 0.01 (strict) based on q-values, considering a maximum deltaCN of 0.05 [22]. Only peptides with minimum peptide length of six amino acids and Rank 1 were considered while the peptide confidence was “medium”. Protein grouping and strict parsimony principle were applied. The results were then exported to an Excel file for further processing.

The spectral count (SpC) values of the proteins identified were normalized using a total signal normalization method. Each sample (i.e., each single sample of cyst fluid collected at different times) was analyzed in two technical replicates with LC-MS/MS. Sequential samples were not pooled, and for each sample the respective master protein list was calculated by averaging the normalized SpC (protein spectral counts) of replicate analyses. In total, 14 LC-MS/MS runs were performed, and 7 master protein lists were obtained: 3 for SM samples, 2 for CS samples and 2 for CG samples. For the differential analysis among tumor histotypes, protein master lists of the samples were averaged in order to obtain a single protein list for each histotype tumor. Among all of the identified proteins, 102 were identified in all samples of at least one tumor histotype. These proteins, named highly reproducible (HR) proteins, were subjected to a label-free quantification approach, as previously reported [23], by applying the differential average (DAve) and differential confidence index (DCI) algorithms [18]. Differentially expressed proteins (DEPs) were selected through the following criteria: upregulated proteins in the first condition have DAve ≥ +0.4 and DCI ≥ +5; downregulated proteins in the first condition have DAve ≤ −0.4 and DCI ≤ −5; the comparisons were SM vs. CS, SM vs. CG and CS vs. CG.

### 2.4. Network Analysis

A PPI (protein–protein interaction) network model per tumor histotype (SM, CS and CG) was reconstructed by considering the HR proteins (n = 102); among HR proteins, both differentially expressed proteins (DEPs, n = 54) selected using Dave/DCI indexes and specific HR proteins (identified specifically in only one histotype: n = 4 for SM; n = 8 for CS; n = 19 for CG) were highlighted in networks. The network models were reconstructed using STRING Cytoscape’s APP (version 3.9.1) [24,25] (http://apps.cytoscape.org/apps/stringapp (accessed on 4 February 2023)), and only protein–protein interactions “databases” and/or “experiments” annotated with a score ≥0.3 and ≥0.15 were retained. The proteins were grouped in PPI functional modules with the support of STRING Cytoscape’s APP [26] and BINGO 2.44 [27]; Homo sapiens organism, hypergeometric 104 test and Benjamini–Hochberg FDR correction (≤0.01) were set.

Among all functional pathways, 13 of them were selected and visualized with a series of pie charts named “Bubble charts” [28]. Each pathway is represented as a circle for each tumor, where the diameter is proportional to the total number of proteins belonging to each pathway. The light blue area of the bubble is proportional to down-represented (or low abundant) proteins while the orange area to up-represented (or highly abundant) proteins within each tumor histotype. To classify a protein as up-represented or down-represented, the SpC values in each tumor were considered: the highest value among them was set as 100, and the others were expressed as relative SpC values. To consider a protein as up-represented, the relative SpC value in a specific cancer histotype had to be equal or higher than the 50% of the reference value; otherwise, the protein was classified as down-represented.

### 2.5. Overrepresentation Analysis

We also submitted the proteins not found in the cystic fluid of all tumors to overrepresentation analysis against all human pathways contained in the database using both the REACTOME (Release 83, https://reactome.org (accessed on 10 July 2022) [29] and PANTHER (Release 17.0; http://www.pantherdb.org (accessed on 2 July 2022) [30] opensource databases in order to identify if any pathways or group of proteins were overrepresented in one tumor. Pathways were considered as significantly enriched in nominal protein set enrichment analysis when *p*-values and false discovery rates (FDRs) were both <0.05.

### 2.6. Statistical Analysis

Pearson’s chi-squared test was used as a test of homogeneity and independence, and two-way ANOVA was used to assess significant differences in protein levels among the different samples, and the level of significance was set to 0.05. All statistical analyses were performed using MedCalc, version 14.8.1 (MedCalc Software, Ostend, Belgium).

## 3. Results

### 3.1. Protein Detected and Reproducibility

We identified, using LC-MS/MS, 1008 different proteins (Supplementary Materials Table S1). We classified the identified proteins as present in serum only (S), in CSF only (CSF), in both serum and CSF (S + CSF) or not previously described in serum or CSF (NS + NCSF) [31,32] (Supplementary Materials Table S1); we found that there were no significant differences in the proportion of these four classes of proteins in the different tumors (χ2 0.483; degrees of freedom (DF), 6; *p* < 0.4830).

Overall, there were no significant differences among the results of the experiments on samples from the same patient obtained at different times (one-way ANOVA: Levene tests for equality of variances, *p* = 0.998, F-ratio: 0.0000000825, *p* = 1.00 for SM; Levene tests for equality of variances, *p* = 0.950, F-ratio: 0.00000168, *p* = 0.999 for CS; Levene tests for equality of variances, *p* = 0.996, F-ratio: 0.0000315, *p* = 1.00 for CG), showing good reproducibility of the results of the proteomic analysis and overall stability in cystic fluids compositions.

**Figure 2:**
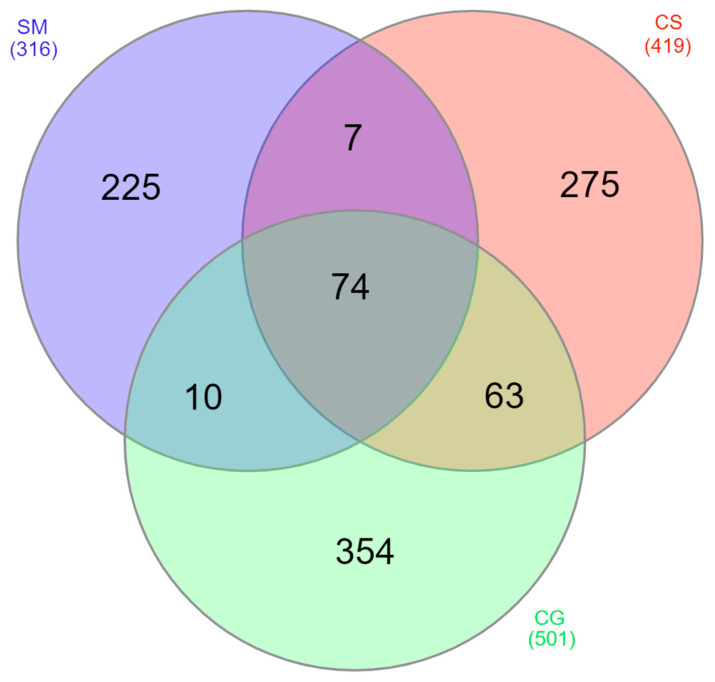
Venn diagram of the distribution among the different tumors of the identified proteins. SM: blue, CS: red and CG: green. Numbers in parentheses refer to the total number of proteins identified in each tumor.

Among the 1008 proteins, 854 were found only in one tumor: 225 in secretory meningioma (SM), 275 in cystic schwannoma (CS) and 354 in cystic high-grade glioma (CG) (Figure 2). Among them, 4 were identified in all samples of SM, 8 in all samples of CS and 19 in all samples of CG (Table 1, Supplementary Materials Figure S1).

Moreover, 49 proteins were present in all tumors and samples tested, and 74 were identified at least once in the cystic fluid of all tumors (Figure 2). As described in the Section 2, we selected 102 proteins present in all samples of at least one tumor histotype, as highly reproducible (HR) proteins (Supplementary Materials Table S2) for further functional evaluation.

### 3.2. Proteins Detected in the Cystic Fluid of All Tumors

The 49 proteins common to all samples of all tumors were also previously identified by deep undeleted proteomic studies in both serum and cerebrospinal fluid (CSF) obtained from normal subjects [31,32]. Among the proteins identified, there were many typical serum proteins, like albumin and immunoglobulins, that, even under normal conditions, enter CSF from plasma but also prostaglandin-H2 D-isomerase, the second most abundant protein in CSF [33], which is predominantly produced by arachnoid cells and oligodendrocytes [34] and appears upregulated in the CSF of gliomas and high-grade meningiomas [35]. Another 25 proteins were common to all tumors and were identified at least once per histotype. Of them, only collagen alpha-5(IV), which was previously documented in plasma, was not described in the CSF of normal subjects [32].

### 3.3. Label-Free Differential Analysis and Systems Biology

**Figure 3:**
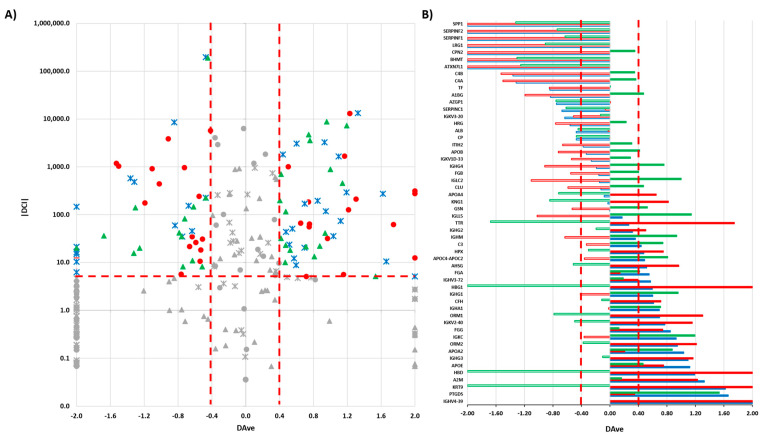
(**A**) Boxplot of DAve vs. |DCI| calculated for all 102 HR proteins in the comparisons of SM vs. CS; SM vs. CG; CS vs. CG. Red circles: SM vs. CS; blue crosses: SM vs. CG; green triangles: CS vs. CG. In grey: discarded proteins according to DAve and DCI filters. (**B**) DAve vs. gene name of 54 DEPs. Red: SM vs. CS; Blue: SM vs. CG; Green: CS vs. CG; Filled (positive) and Empty (negative): up- and downregulated, respectively, in the first condition.

We submitted the 102 HR proteins (Supplementary Materials Table S2) to a label-free differential analysis using the DAve and DCI algorithms [18,23] and by comparing the three different tumor histotypes (SM vs. CS; SM vs. CG; CS vs. CG). Using threshold |0.4| and |5|, respectively, for Dave and DCI, 54 differentially expressed proteins (DEPs) were extracted from HR proteins (Supplementary Materials Table S3) (Figure 3).

**Figure 4:**
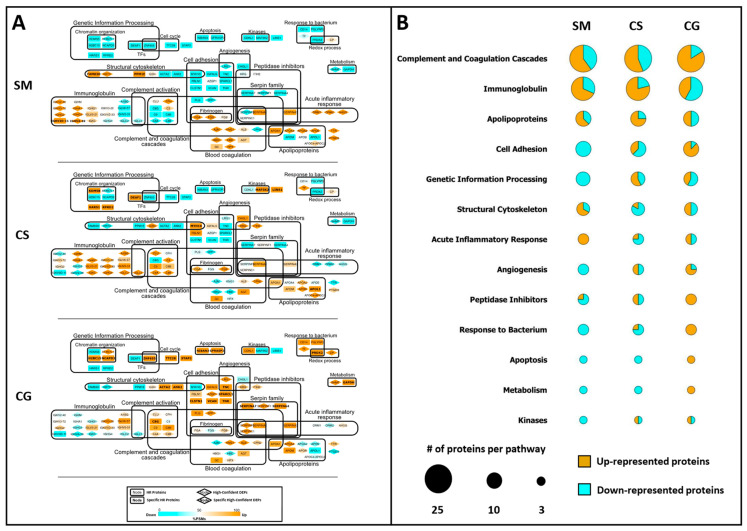
Systems biology analysis of HR proteins. (**A**) Functional PPI networks of HR proteins: nodes with rhombus shape indicate differentially expressed proteins (DEPs), while bold gene names highlight proteins specifically identified in SM, CS or CG. The node color scale (%PSM) shows down- (light blue) and up-represented (orange) proteins. (**B**) Protein expression per functional pathway in SM, CS and CG. Bubble size is proportional to the number of proteins in each pathway; the light blue area is proportional to the number of low abundant proteins, while the orange area is proportional to the number of high abundant protein. Please refer to Section 2.4 (Materials and Methods) for further details.

We also submitted the HR proteins to a systems biology analysis with the aim of identifying functional pathways that both describe the complexity of biofluids and, potentially, highlight some peculiarities in the composition of each cystic fluid. For each tumor histotype, a PPI network was constructed (Figure 4A). Each protein is represented through a node containing the protein gene name; DEPs are indicated by a rhombus node, while tumor-specific HR proteins are highlighted with the gene name in bold. HR proteins were grouped into different functional pathways, according to STRING Cytoscape’s APP and BINGO 2.44 functional annotations. A color graduation scale, from light blue to orange, is attributed to each node according to the relative abundance of the protein in tumors: the highest value of SpC associated to the same protein in SM, CS and CG is set to 100 (orange node), while the other SpC values are expressed as a relative percentage and, consequently, represented as the corresponding colors in the figure’s legend.

In order to simplify the visualization of involved pathways (Figure 4A), the low (blue) and high (orange) abundant proteins for the main 13 functional categories are summarized in the bubble chart, where the diameter is proportional to the number of proteins selected for each pathway (Figure 4B). As shown in Figure 4B, the cystic fluids collected from the different tumors differ in the proportion of up- and down-represented proteins for each pathway considered.

SM contains the highest number of low abundant proteins for the functional terms “Cell Adhesion, “Genetic Information Processing”, “Angiogenesis”, “Response to Bacterium”, “Apoptosis”, “Metabolism” and “Kinases”. Moreover, SM has the highest number of high abundant proteins for the pathway annotated as “Acute Inflammatory Response”.

Conversely, CG tumor displays a high number of up-represented proteins for almost all of the pathways mentioned above, except for the “Acute Inflammatory Response” and “Genetic Information Processing” terms, where the ratio between high and low abundant proteins is balanced. Among the 15 proteins that can be annotated for the pathway “Cell Adhesion”, 5 are specific to CG, although not differentially expressed according to the DAve and DCI algorithms; similarly for the pathway “Apoptosis”, the two proteins annotated for this pathway are exclusive to CG (Figure 4A).

Finally, CS seems to have a protein composition that differs from the other two tumors, with more complex ratios between up- and down-represented proteins per each pathway, except for the “Apoptosis” and “Metabolism” terms, where the low abundant proteins are predominant. Remarkably, among the eight proteins annotated for the pathway “Genetic Information Processing”, four are exclusively identified in CS, representing the 50% of the total number of exclusive proteins identified for this tumor histotype (Figure 4A).

### 3.4. Levels of Specific Immunoglobulin Are Different in Cystic Fluid from Different Tumors

**Figure 5:**
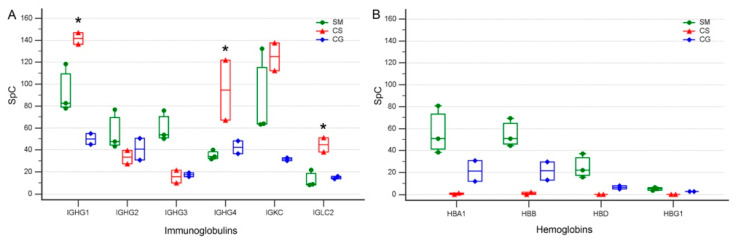
Multiple comparison graphs of the spectral count (SpC) levels of selected proteins present in the cystic fluid of various tumors. (**A**) Gamma immunoglobulin heavy and light chains from SM: secretory meningioma; CS: cystic schwannoma; CG: cystic high-grade glioma. Boxes represent values from the lower to the upper quartiles; vertical lines connect minimum and maximum values; experimental points derived from cystic fluid of SM are represented as green circles, red triangles are those derived from CS, and blue rhombuses are those derived from CG, asterisk indicates immunoglobulin values in CS significantly different from the other tumors (*p* < 0.05 ANOVA). IGHG1: immunoglobulin heavy constant gamma 1; IGHG2: immunoglobulin heavy constant gamma 2; IGHG3: immunoglobulin heavy constant gamma 3; IGHG4: immunoglobulin heavy constant gamma 4; IGKC: immunoglobulin kappa constant; IGLC2: immunoglobulin lambda constant 2. (**B**) Levels of hemoglobins, a marker of blood contamination. Boxes, lines and symbols are as in (**A**). HBA1-2: hemoglobin subunit alpha; HBB: hemoglobin subunit beta; HBD: hemoglobin subunit delta; HBG1: hemoglobin subunit gamma-1.

Immunoglobulins are among the most abundant proteins present in serum and, not surprisingly, the LC-MS/MS analysis we performed on the cystic fluid of three different tumors led to the identification of 38 different immunoglobulin sequences of which 18 were among the 102 HR proteins (Supplementary Materials Table S2). When we consider the gamma immunoglobulins γ1, γ2, γ3 and γ4 (IGHG1, IGHG2, IGHG3 and IGHG4) together with the two light chains (IGKC and IGLC2), which were the most represented immunoglobulins in the cystic fluid of all of the tumors and samples, we found that they resulted as DEPs according to the Dave and DCI algorithms, indicating that their levels significantly differed in the cystic fluid from the different tumors. Indeed, the γ1, γ4 and λ (IGLC2) light chains had remarkably higher levels in the cystic fluid from the CS compared to the SM and CG tumors, while the γ3 heavy chain had a higher level in SM (Figure 5A). These differences were maintained in all samples that were obtained at different times from the same tumor and, at least for CS, cannot be explained by an increased contribution of blood and serum to the cystic fluid in the samples obtained from the CS compared to that obtained from the other tumors, since all hemoglobin isoforms, which are good markers of blood contamination, were actually lower in all samples derived from CS compared to those of the other tumors (Figure 5B). Albumin, a good marker of serum contamination, was higher in CG compared to CS (Supplementary Materials Table S1).

### 3.5. Protein Overrepresentation Analysis

We submitted the proteins not found in the cystic fluid of all tumors, to overrepresentation analysis in order to identify if any pathways or group of proteins were overrepresented in one or more tumors. In Supplementary Materials Table S4, we report the REACTOME pathways resulting from overrepresentation analysis associated with both *p*-values and the false discovery rate (FDR) inferior to 0.05. We found both overrepresented pathways specific to each tumor and pathways common to more than one tumor. It is of note that some of the overrepresented pathways in specific tumors such as keratinization (R-HSA-6805567) in SM are highly compatible with what is already known for this peculiar variant of meningioma [36,37].

## 4. Discussion

Proteomic characterization of cystic fluid from brain tumors has been rarely attempted [38,39], and to the best of our knowledge, no studies of multiple samples from different cystic tumors are present in the literature.

Our bottom-up proteomic approach to the proteins contained in the cystic fluid collected at multiple times from the same SM, CS and CG cystic fluids allowed for the identification of over one thousand proteins. Moreover, since all of our samples were collected through a minimally invasive approach and not at or close to the time of an open surgical approach to the tumor, our results should be free of possible artifacts linked to open surgical manipulations. The most abundant proteins, albumin, certain apolipoproteins and immunoglobulins, identified in all samples of all tumors, are derived from plasma, although they are also present at a lower level in CSF [32]. Other typical plasma proteins we found in the cystic fluids, haptoglobin, fibrinogen, transferrin, and alpha 2-macroglobulin can also be produced by the brain itself [40]. However, the contribution of local production to the CSF levels of these proteins compared to the amount of the same proteins derived from plasma is minimal [41].

These findings support the current pathogenetic hypothesis that cyst formation in brain tumors is a consequence of the local disruption of the blood–brain barrier (BBB) leading to the exudation of plasma proteins and their accumulation in cystic fluid [4,5]. However, we also identified in all of our samples, albeit at different levels, prostaglandin D2 synthase, a protein produced by the arachnoid cells that is considered to be a very reliable clinical marker of CSF presence [33,34]. This suggests a mixed origin of all cystic fluid in our samples from both plasma and CSF.

The permeation of CSF by plasma-derived proteins in humans varies according to developmental stages and pathological conditions. Under normal conditions, permeation is maximal during fetal and early postnatal life [42]. All of the cystic fluids we analyzed contained significantly increased concentrations of plasma proteins suggesting that, as is the case with many metabolic and biochemical pathways found in tumors, the accumulation of cystic fluid may involve the reactivation of permeation and secretory processes that under normal conditions are limited to development. Albumin transfer from blood to the CSF is tightly controlled and the albumin-binding glycoprotein, secreted protein acidic and rich in cysteine (SPARC), is instrumental for the increased transfer of albumin [43] to the CSF. We found that the albumin concentration in the cystic fluid was maximal in glioma samples, but albumin was also one of the most abundant proteins in the cystic fluids collected from SM and CS. SPARC protein is secreted by astrocytes [44] and expressed by gliomas and malignant meningiomas, but in these last tumors its role has been recently challenged [45]. We did not identify SPARC in the cystic fluid of any of the studied samples, but we found SPARC-like 1 (SPARCL-1), a paralogue of SPARC, in the fluid collected from CG. This protein, which phylogenetically shares many of the functions of SPARC [46], was also identified in another proteomic study of cystic fluid derived from high-grade glioma samples [38]. Moreover, SPARCL-1 expression has been previously associated with gliomas [47] and the proliferative potential of glioma cancer stem cells [48]. Another route for plasma proteins to enter the cystic fluid may be represented by the damage of small vessels present on the cystic wall. In the cystic fluid of at least one sample of all tumors, we found collagen alpha-5(IV) chain (COL4A5). This protein is an important component of vascular structures like the kidney glomerulus and the stria vascularis of the inner ear [49] and, to a lesser extent, of brain vessels [50]. The presence of this protein in the cystic fluid may suggest the presence of chronically damaged vessels that release it. Chronic damage may lead to abnormal vascular permeability with leakage of serum proteins into the cystic fluid, similar to what occurs in Alport syndrome, where the genetic inactivation of COL4A5 in glomerular vessels leads to increased proteinuria [49]. However, simple leakage mechanisms alone cannot explain all of the differences in the levels of serum proteins we found in the different cystic fluids; IgG, one of the most abundant isotypes in serum and CSF, showed large quantitative differences in isotypes among the cystic fluids of the three different tumors studied, and these differences were maintained in all samples from the same tumor. IgG heavy-chains γ1 or γ4 and light-chains λ constant 2 were significantly enriched in the samples of the cystic fluid of CS compared to those of the other histotypes. These differences cannot be explained by increased blood contamination of the CS samples compared to those derived from other tumors, since all hemoglobin isoforms that are indicative of the extent of blood contamination were actually lower in samples derived from CS. A further indication that passive mechanisms alone cannot explain the difference in the levels of immunoglobulins in the cystic fluid of different tumors is our finding that not all immunoglobulins were higher in the CS cystic fluid compared to that of the other tumors, e.g., IgG heavy-chains γ3 were higher in SM.

In general, differences in the content of specific immunoglobulins between different tumors suggest differences in local host immune function in the tumors studied. These variations may be due to differences in retention or clearance of the antibodies in the different tumoral cyst investigated or to local antibody production by B lymphocytes contained in the cystic fluid of CS. Of note, higher concentrations of immunoglobulins in the cystic fluid of glioblastoma compared to serum have been considered as an index of an ongoing immune response triggered by the tumor [51]. Although further studies will be necessary to distinguish among the above mechanisms, our findings indicate that the level of plasma derived immunoglobulins in the cystic fluid of the tumors is not solely explained by purely passive mechanisms.

Among HR proteins, we identified 54 proteins differentially expressed (DEPs) in at least one of the three comparisons SM vs. CS, SM vs. CG and CS vs. CG by the label-free differential analysis, using DAve and DCI algorithms. Moreover, the PPI network of the HR proteins, together with their label-free quantification, allowed the identification of tumor specific pathways. In SM, two functional pathways “Structural Cytoskeleton” and “Acute Inflammatory Response” emerged as overrepresented, while “Genetic Information Processing” was overrepresented in CS, and “Cell Adhesion”, “Angiogenesis” and “Peptidase Inhibitors” were overrepresented in CG.

We detected in the cystic fluid of all tumors some proteins that are already known to play a role in one or more of the cystic tumors studied: gelsolin (GSN), haptoglobin (HP), alpha-2-HS-glycoprotein (AHSG) and alpha-1-antichymotripsin (AACT). GSN and HP are proteins present in plasma, but they also play a role in meningiomas [52] and gliomas [53], where GSN is downregulated. AHSG is secreted and acts as peptidase and kinase inhibitors involved in the acute-phase response; it is present in the perilymph of vestibular schwannoma patients, and its level is significantly correlated with the insurgence of hearing loss [54,55]. The same protein, also known as fetuin A, may be produced by human glioblastoma cells and, at least in vitro, its knockdown reduces their motility and invasive capacity [56]. AHSG has also been detected in the cystic fluid of adamantinomatous craniopharyngioma where it may play a role in the deposition of calcium [16]. AACT is also an abundant component of the cystic fluid collected from adamantinomatous craniopharyngioma [57,58] another cystic intracranial tumor that originates from the craniopharyngeal duct [59] and not from derivatives of neuroepithelium or neural crest such as the intracranial cystic tumors we investigated.

Different levels of apolipoproteins were present in cystic fluids from all tumors. Although the role of apolipoproteins is very important in lipid metabolism outside the CNS, some of them play an important role in neurodegenerative disease and brain tumors. Clusterin/apolipoprotein J (CLU) is a secreted chaperone that has been reported as a proteomic marker associated with meningiomas [52]. CLU was present at various concentrations in the cystic fluid of all tumors tested and its level was significantly higher in the schwannoma samples. CLU and apolipoprotein E (APOE) are also instrumental in transferring long chain saturated lipids produced in reactive astrocytes to neurons, where they modulate neuronal survival and mediate astrocyte-induced toxicity after CNS injury and disease [60]. Apolipoprotein L1 (APOL1) is found in the CSF of patients affected by frontotemporal lobe dementia [61], and in our samples it was present only in CS. Apolipoprotein D (APOD), present in at least one sample of all tumors, increases upon brain aging and neurodegeneration, and it acts by controlling the redox state of cellular and extracellular lipid structures. APOD is associated with specific subtypes of detergent-resistant microdomains in astrocytic membranes and may also interact with neuronal membranes. APOD associated microdomains contain plasma membrane and endosomal-lysosomal compartment lipid raft proteins [62].

Many proteins we identified at low or intermediate levels in the cystic fluids were not common to all tumors indicating that some proteins derive from tumor cells and/or neural, glial and reactive cells that surround the cystic wall of the tumors by various mechanisms such as secretion or shedding.

A typical example is represented by the numerous cytokeratins we identified in the cystic fluid of SM. SM often shows at the immunohistochemical level keratin expressing cells [36,37]. In the same samples we also identified a scaffold protein: family with sequence similarity 83 member E (FAM83H), that recruits casein kinase 1 (CK1) which is important in keratin cytoskeleton organization [63]. Consistent with the above findings, a network analysis identified structural cytoskeleton and protein overrepresentation analysis identified keratinization as one of the functional pathways associated with SM. Network analysis also identified acute inflammatory response as an overrepresented pathway in SM. Proteins like AHSG, alpha-1-acid glycoprotein 1 (ORM1) and alpha-1-acid glycoprotein (ORM2) present in cystic fluid of SM are related to this pathway. Inflammatory cells are present in meningiomas and correlate with peritumoral edema, growth and aggressiveness [64]. A2M (alpha-2-macroglobulin) an extracellular protease inhibitor involved in coagulation, inflammation and immune response [65] was also more abundant in SM cystic fluid. Interestingly, increased A2M was previously described as a biomarker of meningioma [66].

In cystic fluid from CS retinol-binding protein 4 (RBP4) was present and in the same specimens we also identified, at lower levels, aldehyde dehydrogenase 1 family member A1 (ALDH1A1), also known as retinal dehydrogenase, an enzyme that plays an important role in retinol metabolism and it is upregulated in schwannomas [67]. Increased levels of vitamin A ester are also present in nerve sheath tumors [68] that are closely related to schwannomas.

We detected in all samples of CS deformed epidermal autoregulatory factor 1 homolog (DEAF-1). DEAF1 downregulate the expression of genes encoding for glycolytic enzymes [69], but it also has a role in the immune response being required for intranodal expression of peripheral tissue-restricted antigens responsible for modulating the acute graft-versus-host disease [70].

Osteopontin (SPP1) a protein that, among other things, is involved in merlin/schwannomin degradation [67] and in enhancing glioblastoma aggressiveness [71] was also present in cystic fluid collected from CS and CG. Protein lines homolog 1 (LINS1) that was also present in all CS samples is a regulator of the wingless/Wnt pathway [72] whose inhibition decrease vestibular schwannoma growth [73].

Among the proteins specific for the cystic fluid of CG that are known to play an important role in gliomas, there were tenascin-R (TNR), histones H2A (H2A2A) and H2B (H2B). Tenascins are glycoproteins that are secreted by immature glia being downregulated in the adult brain unless it is injured or affected by inflammation or neurodegeneration. TNR is expressed by all gliomas and its level varies inversely with malignancy [74,75]. Histones are known to be released by injured or dying cells [76] in all contexts, however histones H2A2A and H2B contain globular protein domains that are frequently mutated in cancers [77] but not in gliomas where histone H3 is most frequently mutated [78]. Extracellular histone H2B is increased in sites of neovessel formation and triggers apoptosis and pyroptosis reducing angiogenesis in vivo and in vitro, this effect is dependent on TLR2/TLR4 [79]. It is also an important component of neutrophil extracellular traps [80] together with protein–arginine deiminase type-4 [81] another protein that we found exclusively in glioma cystic fluid. Neutrophil extracellular traps (NETs) have immune-modulatory functions and can be associated with sterile inflammation and promote thrombosis causing small vessel occlusion [82]. Level of H2B correlates with proliferative response after radiation therapy in gliomas [83] and antibodies against H2B are detectable in CSF of multiple sclerosis patients [84].

Other components of cystic fluid from CG were kininogen (KNG1) and leucine-rich alpha-2-glycoprotein 1(LRG1) that belongs to the functional pathway angiogenesis that is enriched in CG. Both these proteins that were previously described in the cystic fluid of high-grade gliomas [38] are implicated in neovascularization.

## 5. Conclusions

Our results suggest that plasma proteins leaking from blood vessels with altered blood–brain barrier are an important contributor to cyst fluid formation, but CSF and the tumor itself also contribute to cystic fluid proteome. Our findings also show that the proteome of the fluid contained in brain tumor cysts is stable over time, and it is possible to identify pathways and proteins that uniquely differentiate these tumors. Some of these proteins were previously identified in serum or in the tumor tissue of patients affected by the same tumors we studied strengthening our conclusion that proteomic analysis of cystic fluid may contribute to the understanding of the host–tumor relationships of cystic brain tumors and their diagnosis.

## Figures and Tables

**Table 1:** Proteins we identified in only one of the cystic tumors and present in all samples of the same tumor. Tu: tumor; SM: secretory meningioma; CS: cystic schwannoma; CG: cystic high-grade glioma; Accession: UniProt accession number; Gene: symbol according to the HUGO gene nomenclature committee; Protein: UniProt recommended name; MW (KDa): molecular weight in kilodalton; pI: isoelectric point.

Tu	Accession	Gene	Protein	MW (KDa)	pI
SM	A0A494C1T9	FAM83H	Protein FAM83H	149	6.71
SM	P01824	IGHV4-39	Immunoglobulin heavy variable 4–39	13.9	9.26
SM	A0A0A0MRZ8	IGKV3D-11	Immunoglobulin kappa variable 3D-11	12.6	5.29
SM	Q8WY54	PPM1E	Protein phosphatase 1E	83.9	5.01
CS	O14791	APOL1	Apolipoprotein L1	43.9	5.81
CS	A0A0J9YWD6	DEAF1	Deformed epidermal autoregulatory factor 1 homolog (Frag.)	17.7	7.11
CS	E7ETE2	HARS1	Histidyl-tRNA synthetase 1	57.8	5.88
CS	Q9UGL1	KDM5B	Lysine-specific demethylase 5B	175.5	6.7
CS	Q8NG48	LINS1	Protein Lines homolog 1	85.8	6.52
CS	Q9Y2U5	MAP3K2	Mitogen-activated protein kinase kinase kinase 2	69.7	8
CS	P35580	MYH10	Myosin-10	228.9	5.54
CS	Q5VT52	RPRD2	Regulation of nuclear pre-mRNA domain-containing protein 2	155.9	7.42
CG	P62736	ACTA2	Actin, aortic smooth muscle	42	5.39
CG	A0A5F9ZHS1	ANK2	Ankyrin-2	436.8	5.16
CG	P07360	C8G	Complement component C8 gamma chain	22.3	8.31
CG	O94985	CLSTN1	Calsyntenin-1	109.7	4.91
CG	P04406	GAPDH	Glyceraldehyde-3-phosphate dehydrogenase	36	8.46
CG	Q96D09	GPRASP2	G-protein coupled receptor-associated sorting protein 2	93.7	5.01
CG	U3KQK0	H2BC15	Histone H2B	18.8	10.54
CG	P42695	NCAPD3	Condensin-2 complex subunit D3	168.8	7.5
CG	Q86XR2	NIBAN3	Protein Niban 3	77.4	8.63
CG	P32119	PRDX2	Peroxiredoxin-2	21.9	5.97
CG	P29622	SERPINA4	Kallistatin	48.5	7.75
CG	P05543	SERPINA7	Thyroxine-binding globulin	46.3	6.3
CG	Q14515	SPARCL1	SPARC-like protein 1	75.2	4.81
CG	Q9UGK3	STAP2	Signal-transducing adaptor protein 2	44.9	8.16
CG	P24821	TNC	Tenascin	240.7	4.89
CG	Q92752	TNR	Tenascin-R	149.5	4.82
CG	Q96AY4	TTC28	Tetratricopeptide repeat protein 28	270.7	6.89
CG	P13611	VCAN	Versican core protein	372.6	4.51
CG	Q8N720	ZNF655	Zinc-finger protein 655	57.4	7.14

## Data Availability

The proteomic datasets (in form of raw data) analyzed for this study can be found in the MassIVE database (massive.ucsd.edu) at the following link: ftp://MSV000091725@massive.ucsd.edu (accessed on 17 April 2023).

## Supplementary Materials

The following supporting information can be downloaded at: https://www.mdpi.com/article/10.3390/cancers15164070/s1, Figure S1: Venn diagram of the proteins present in all samples of only one tumor; Table S1: All proteins identified with LC-MS/MS; Table S2: List of the highly reproducible (HR) proteins; Table S3: Results of a label-free differential analysis on the 102 HR proteins using the DAve and DCI algorithms and by comparing the different tumors; Table S4: Pathways resulting from overrepresentation analysis using REACTOME and PANTHER.
