# Supplementary figures and images for: Proteomic Analysis on Sequential Samples of Cystic Fluid Obtained from Human Brain Tumors
Source: Cancers (Basel). 2023 Aug 11;15(16):4070. doi: 10.3390/cancers15164070 (PMC10452907; doi:10.3390/cancers15164070)

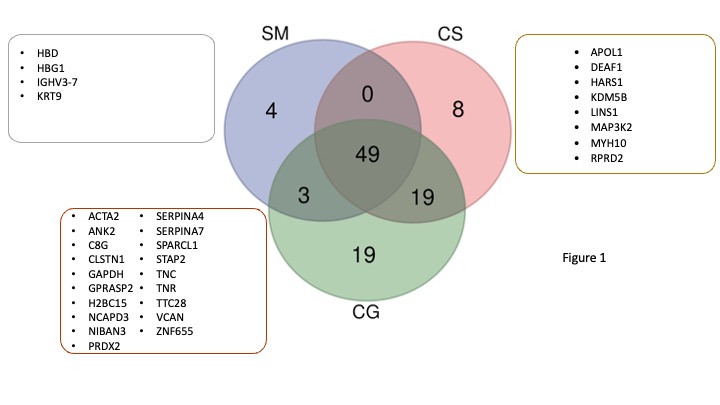

Supplement: Supplementary file 1 [file cancers-15-04070-s001.zip › Figure S1.jpeg]
